# A review of utilization of waste polyurethane foam as lightweight aggregate in concrete

**DOI:** 10.1016/j.heliyon.2024.e40479

**Published:** 2024-11-17

**Authors:** R. Roobankumar, M. SenthilPandian

**Affiliations:** School of Civil Engineering, Vellore Institute of Technology, Chennai Campus, Chennai, 600127, India

**Keywords:** Polyurethane foam waste, Waste management, Flexible polyurethane foam, Rigid polyurethane foam, Lightweight aggregate concrete, Fresh concrete properties, Hardened concrete properties, Microstructure characterization

## Abstract

Engineered concrete mixes using industrial waste as a construction material are an enormous step towards sustainable development and financial benefits. The refrigeration, automobile, and construction industries mainly generate polyurethane foam waste material. Most of the polyurethane foam wastes are dumped in landfills or incineration, which creates environmental effects. Polyurethane foam waste is challenging to recycle because of its bulky nature, limited recycling methods, high transportation costs, complex chemical composition, and inadequate collection and processing infrastructure. Utilizing waste polyurethane foam as lightweight aggregate in concrete serves a dual purpose: reducing natural aggregate extraction and reducing polyurethane foam waste going to landfills. This article reviewed waste disposal rigid polyurethane foam as a lightweight aggregate in concrete mixtures. Initially, it discusses the statistical data analysis, physical and microstructural properties of waste flexible and rigid polyurethane foam. After that, performance evaluations focused on fresh properties by slump tests, hardened properties by compressive strength and density, and microstructure analysis by scanning electron microscopy are presented. The study concludes that incorporating waste polyurethane foam increases workability, improves bonding between polyurethane foam aggregate and the cementitious matrix, and reduces concrete density and compressive strength for lightweight concrete structures. This paper discusses the benefits of utilizing solid waste rigid polyurethane foam in concrete compared to conventional concrete. This study also identified the research gaps in the current state of knowledge and provided few recommendations for future research work.

## Highlights

• Statistical data analysis of PU foam production, waste disposal, and type of waste were discussed.
• The physical properties of PUFW as coarse aggregate were reviewed.
• The fresh and hardened concrete properties of LWAC with the incorporation of PUFW were reviewed.
• Microstructure analysis of the PU foam aggregate and PU foam aggregate concrete was discussed.

## 1 Introduction

The demand for concrete has increased daily, owing to the increasing population growth rate. Therefore, there is a great need to construct more residential buildings and infrastructure. Every year, there is a growing demand for traditional building materials such as cement, sand, and aggregates used in the building industry [1]. Traditional building materials increase the cost of constructing a new house, increase the weight, and delay the completion of work [2,3]. Mining worthy sand from river beds for construction is a serious problem because it causes flooding or transfers water flow. Thus, few states and countries have banned sand mining. Natural stone reserves have substantially decreased, and environmental harm has resulted from the rising demand for concrete in the construction industry using normal-weight aggregates (NWA) [4]. Researchers are concentrating their research on using waste or recycled material in construction due to the increasing demand for sustainable development. So, there is a time requirement to look for another sustainable material (waste, recycled, or lightweight) for aggregates. Lightweight aggregate (LWA) of low bulk density can be classified into natural and artificial LWAs [5]. The previous studies report that different types of artificial LWAs in concrete, such as sintered fly ash [6], Expanded Polystyrene beads [7], crushed coconut shells [8], crushed brick and stone [9], oil palm shells [10], expanded perlite [11], expanded clay and shale [12]. The main natural LWAs discussed are sawdust [13], palm oil clinker [14], stone-sawing mud [15], volcanic ash [16], recycled aggregate [17], volcanic pumice [18,19], boron waste [20], diatomite and scoria [21]. According to the American Standard Testing Methods (ASTM), LWA must have a bulk density of less than 1040 kg/m^3^ for a mixture of fine and coarse material, less than 1120 kg/m^3^ for fine aggregate, and less than 880 kg/m^3^ for coarse aggregate [22]. As per the Indian Standard (IS) code [23] combined aggregates dry, loose bulk density shall not exceed 1100 kg/m^3^. This European Standard (ES) code [24] shows LWA as of quartz origin with particle densities not more than 2000 kg/m^3^ or loose bulk densities not more than 1200 kg/m^3^.

Lightweight Aggregate concrete (LWAC) mixture is produced with a lightweight coarse aggregate instead of normal aggregate [25,26]. Lightweight concrete has recently emerged in the construction industry. It has many advantages over traditional concrete, including lower construction costs and the ability to complete work quickly [27,28]. Researchers have carried out studies on the use of various waste by-products (construction industry waste, agriculture waste, miscellaneous and natural waste, quarrying dust waste, recycled plastic, and hazardous waste) as aggregates, sand, admixtures, cement and their combinations in LWAC for sustainable development, recycling, and environmental production [[29], [30], [31], [32]]. LWAC is helpful because it reduces the weight of structural components by more than 20 % [33]. According to the IS Code [34], typical concrete has a density of about 2400 kg/m^3^ and is regarded as strong, resilient, and heavy. This problem arises with the introduction of contemporary technologies like precast concrete, precast wall panels, precast deck slabs, prefabricated concrete, and off-site buildings. Building precast structures necessitates the use of trucks with a significant lifting capacity. More giant cranes and trucks are required, which increases the financial burden and adversely affects the environment. This challenge can be reduced by implementing concrete mixtures with lightweight materials [35]. Lightweight concrete has been developed and is widely used in construction applications to reduce the weight of structures. It has good thermal insulation characteristics, sufficient load-bearing resistance, robustness, and energy savings [36,37]. The IS [38] code specifies the LWAC density as not exceeding 1000 kg/m^3^. According to the American Concrete Institute (ACI 213R) [39], LWAC has a compressive strength of at least 17 MPa and a density between 1350 and 1900 kg/m^3^. The British Standard (BS) code [40] calls for LWAC with compressive strengths between 8 and 80 MPa and densities between 800 and 2000 kg/m^3^.

Polyurethane (PU) foam is widely used in various industries, including building industries for thermal and acoustic insulation and automotive industries for reducing vehicle weight. In addition, there is a demand in the refrigeration industry for insulation on refrigerator and freezer walls and doors [41]. Most countries use polyurethane foams in pipeline construction to minimize soil erosion [42]. There are two main types of PU foams: flexible PU foam, which has better qualities such as excellent elasticity, flexibility, elongation, chemical stability, and solvent resistance. Flexible foam is widely used across various industries due to its cushioning, insulating, and flexible properties. In footwear, flexible foam is primarily used for cushioning and support. It forms the base of insoles, providing comfort and shock absorption for the feet, especially in athletic and casual shoes. In upholstery, flexible foam is a key material in the padding of furniture such as sofas, chairs, and cushions. The automotive industry utilizes it in car seats, headrests, and insulation for noise reduction [43,44]. Another main type is rigid PU foam, which has significant qualities such as excellent mechanical performance, resistance, insulation, and sound absorption [45]. Rigid foam is widely used for insulation, packaging, and structural support. In construction, it's used in walls, roofs, and floors for thermal insulation and energy efficiency. It also forms part of Structural Insulated Panels (SIPs). In the automotive and aerospace industries, it provides lightweight insulation and structural components [46,47]. Millions of tonnes of PU foam waste are generated from manufacturing leftover materials, the destruction of insulation panels used in the construction industry, scrapped car seats, and the end of their life cycle [48]. Most of the waste PU foam is disposed of in landfills and incinerated [49]. The burning of PU foam waste creates severe fire hazards, emits potentially harmful compounds, and may contribute to smog, which has become a severe environmental issue. Landfilling with PU foam waste reduces the number of valuable sites available [50]. With a growing worldwide awareness of environmental protection and the idea of sustainable development, substantial changes in environmental issues, and the growing need for a clean and green earth, the demand for PU foam reuse has been increasing [51].

Recently, Somarathna et al. [52] review article discussed the physical and mechanical properties of PU foam and the use of PU foam for structural and infrastructural engineering applications in terms of composites, protective coatings, and strengthening. The review did not thoroughly evaluate the physical properties and microstructure analysis of waste PU foam as fine and coarse particles for LWAC, and the mechanical properties of PU foam lightweight aggregate concrete were reviewed using polyurethane for structural and infrastructural engineering applications. That review article discussed. In another review, X. Yuan. et al. [53] studied the fundamental analysis of PU foam, foaming methods, the fresh properties of the foamed concrete, and its durability properties. However, the review did not establish the statistical data analysis of PU foam production, waste disposal and type of waste, the mix proportions, and the workability of lightweight concrete with waste PU foam as aggregate. In addition, the review did not elaborate on published results on the compressive strength and density of lightweight concrete with the incorporation of PU foam waste as aggregate.

To address these gaps in the literature, a thorough evaluation is required to examine the acceptability of various types of waste PU foam materials as partial and complete substitutes for fine and coarse aggregate in lightweight concrete. Ben Fraj et al. [54] used waste rigid PU foam (8–20 mm in size and a density of 45 kg/m^3^) as coarse particles in LWAC to develop compressive strength with the required density. Mounanga et al. [55] investigated 0–10 mm size of waste rigid PU foam with a density of 45 kg/m^3^ in the concrete mixture to develop the mix proportions, mechanical performance and microstructure analysis. According to Wang et al. [56] waste rigid PU foam (8–20 mm) with a density of 45 kg/m^3^ was used for coarse particles in concrete to develop compressive strength and microstructure. In another research, Vaclavik et al. [57] stated that adding waste PU foam (0–6 mm) with a density of 30–60 kg/m^3^ as a fine aggregate in lightweight concrete to develop mechanical performance. Furthermore, Tomas et al. [58] reported a new finely ground limestone (FGL) instead of sand with a rigid PU foam waste (4–8 mm) with a density of 35 kg/m^3^ in the concrete mixture to develop the mechanical performance and workability of lightweight concrete. Gómez-Rojo et al. [59] discussed the physical properties of waste PU foam as an eco-efficient building material. The mentioned researches the idea of using waste disposal PU foams as fine and coarse aggregates for making LWAC. This article reviewed waste disposal PU foam as a lightweight aggregate in cement concrete mixtures. Initially, it discusses statistical data analysis of PU foam production, waste disposal, type of waste and recycling. After that, the physical properties and microstructure analysis of waste PU foam for lightweight concrete. Furthermore, lightweight concrete mix proportion, workability, and mechanical performance are incorporated with waste PU foam as a fine and coarse aggregate.

Using waste disposal PU foam in cement concrete mixtures is important because it effectively diverts waste from landfills and reduces demand for traditional materials such as aggregate and sand, conserving natural resources and reducing the environmental effects of extraction and processing. Incorporating waste PU foam into concrete mixtures can provide financial benefits by lowering material costs, as PU foam, being a waste material, is generally cheaper than traditional materials. Significant savings can also be achieved through reduced transportation and disposal costs in waste management. Since PU foam is lighter than traditional aggregates, it reduces the overall density of the concrete, resulting in lighter structures. This is especially beneficial in applications where weight reduction is important, such as in floors and roofing. Polyurethane foam concrete offers economic benefits in construction due to its lightweight, reduced material and transportation costs, and excellent thermal insulation, which lowers long-term energy expenses. While the initial cost of polyurethane foam may be higher, savings in labor, faster construction times, and long-term energy efficiency can offset these costs. Its economic viability depends on project needs, but in cases where insulation, reduced weight, and efficiency are priorities, it can be a cost-effective solution.

## 2 Polyurethane (PU) foam

### 2.1 Origin of PU foam

Wurtz produced the first urethane in 1849 [60]. Following that, Otto Bayer, Germany, produced PU in 1937 by reacting a polyester diol with a di-isocyanate [61]. PU foam is another large polymer product group that includes mainly flexible PU foam and rigid PU foam [[62], [63], [64]]. The first one is flexible PU foam with an open cell structure, which is classified based on the method of synthesis and the polyol used [44,65,66]. The second one is rigid PU foam with a closed cell structure, which was introduced to the market in 1967 due to its increased thermal stability and flammability resistance [63]. No heating is required to create rigid PU foam at room temperature [67,68]. Flame resistance refers to the ability of a material to withstand ignition and prevent the spread of fire. In the context of rigid PU foam, flame resistance is critical for safety, as it can help reduce fire hazards, protect property, and enhance occupant safety in buildings and vehicles [61,69]. Spray and component foaming are two methods to produce this rigid PU foam. Spray foaming is a highly effective method for applying rigid PU foam directly onto surfaces, such as in attics or wall cavities. When sprayed, the foam expands rapidly upon contact with air, filling gaps and adhering to the substrate, thereby creating a seamless insulation barrier. This method is particularly advantageous for complex shapes and irregular surfaces, as it provides excellent thermal insulation, air sealing, and moisture resistance [70]. The two-component foaming method involves mixing two key components, polyol and isocyanate, in a precise ratio. Pouring is another effective method used to produce rigid PU foam, particularly in the creation of large panels or blocks. In this process, the mixed components are poured into molds where they expand and cure [71]. Injection molding is a technique used to create rigid PU foam products with complex shapes and high precision. In this method, the foam mixture is injected into a closed mold under pressure. The rapid expansion of the foam fills the mold completely, resulting in uniform products such as blocks or specific components for automotive applications [72].

### 2.2 PU foam waste

According to the latest Global Polyurethane Market Volume 2015–2030 report published by the Statista research department, the global polyurethane market volume was estimated to be nearly 26.22 MMT in 2023. It was expected to increase to 31.27 MMT in 2030 [73]. The demand for PU foam is approximately 25 % in construction and building, 20 % in automotive, 25 % in refrigeration, and 30 % in textile and other industries. Around 30 % of PU foam waste is generated annually from the estimated total market volume, of which 33 % is recycled, 45 % is incinerated, and 22 % is disposed of in landfills [59]. PU foams are classified into two major types based on their densities: flexible and rigid foam [74]. Waste PU foam is mainly generated from manufacturing leftover materials, unshaped panels, and product scraps. Additionally, PU foam waste is generated from destroying insulation panels used in the building industry and when furniture is discarded [75]. Recycling PU foam waste can help the environment by decreasing the need for traditional materials and redirecting waste away from landfills and incineration facilities [76]. However, PU foam recycling can be difficult due to the complicated composition of the material and inefficient recycling processes. Additionally, the recycling process may require energy and resources [77]. Incinerating PU foam waste can emit hazardous chemicals and pollutants into the atmosphere, causing air pollution and harming human health. Furthermore, incineration may not destroy all waste and may leave ash leftovers that must be disposed of separately [78].

### 2.3 Disposal of PU foam waste

Most PU foam waste is disposed of in landfills, which takes up valuable space and may produce dangerous chemicals as it decomposes, adding to long-term environmental damage. Landfill disposal of PU foam waste involves collecting discarded foam from various sources, such as manufacturing facilities and consumer products, and transporting it to designated landfill sites for permanent disposal. While this method is straightforward, it raises significant environmental concerns. PU foam is not biodegradable and can take hundreds of years to decompose, contributing to landfill space depletion. Additionally, the breakdown of PU foam may lead to the leaching of harmful chemicals into soil and groundwater, posing risks to ecosystems and human health. To address these issues, it is essential to explore alternative disposal methods, such as recycling and public awareness initiatives, to reduce the volume of PU foam waste that ends up in landfills [49,79]. The Netherlands, New Zealand, Sweden, Denmark, and Switzerland have recently passed regulations restricting the use of land disposal [80,81]. Germany and Australia currently restrict the disposal of materials with a high carbon content [78]. On the other hand, incineration involves burning waste PU materials for heat recovery. Burning 1 kg of PU can yield 7000 kcal/kg of calorific value, producing heat equivalent to that produced by the exact weight of coal. Burning can reduce the volume of garbage by 97 % [82]. Heejoon Kim created eco-fuel by combining PU foam waste with coal to create an innovative and sustainable way to dispose of the waste [83].

### 2.4 Recycling of PU foam waste

There are two major recycling methods for PU foam waste: physical and chemical recycling [48,80]. Physical recycling, which involves reusing PU wastes without chemical treatment, is easy, affordable, practical, and environmentally friendly [84,85]. Grinding and regrinding are two key physical recycling methods for polyurethane foam. In grinding, PU foam is shredded into smaller particles, which are then used as filler material or bonded together to create rebonded foam products like carpet underlay or insulation. In regrinding, the foam is reduced to a fine powder and mixed with virgin polyurethane to produce new foam products, particularly for low-density applications such as cushions [86]. Chemical recycling involves reactions including glycolysis, hydrolysis, amino-lysis, thermochemistry, and biodegradation methods [87,88]. The main objective of recycling is to get back to the original raw materials, especially to make high-quality polyol monomers from recovered polyol monomers [89,90]. Flexible and rigid polyurethane foams are used as fillers in applications like furniture, carpet underlay, packaging, construction materials, and automotive components. Flexible foam is common in cushions and protective packaging, while rigid foam is used in insulation panels and lightweight building materials. Using PU foam waste as fillers is beneficial because it reduces material costs, minimizes landfill waste, saves energy, and enhances product properties like insulation and shock absorption. This makes it a sustainable and cost-effective recycling option [59]. Few researchers have used recycled PU foam in mortar, concrete, and plaster. Junco and Gadea [91], used recycled PU foam from the refrigeration industry with particles up to 6 mm in mortar to replace conventional small-size aggregate. They investigated A salt spray test, a sulphur dioxide test, a Kesternich test, and a hot water resistance test are among the ageing tests to assess the toughness of recycled mortars and suggest that up to 100 % of the aggregate in this type of masonry mortar can be replaced [[91], [92], [93]]. Molero et al. [94], studied the plaster mixtures made with recycled PU foam for consistency, workability, and mechanical properties. The result shows that increasing the PU foam waste quantity decreases the density and mechanical properties. Thus, this plaster PU foam is for thermal and sound insulation.

## 3 Physical properties of PU foam

In this study, after converting the disposal of PU foam waste to aggregate particles, the physical properties of PU foam aggregate are reviewed: apparent density, real density, water absorption, porosity, shape, thickness, surface texture, compressive strength, and microstructure analysis. Fragmenting PU waste foam is challenging due to its lightweight, porous, and low-density nature. Researchers use shredding machines to break the foam into smaller pieces, followed by grinders to further reduce the size for specific applications, such as in concrete production [59,95]. Rigid waste PU foam from construction, can be reused in concrete, but exposure to aging and ultraviolet (UV) radiation may affect its performance. UV radiation can cause brittleness, surface cracking, and oxidation, weakening the PU foam and potentially reducing the strength of concrete made with it. Aging can also lead to changes in density, porosity, and moisture absorption, further impacting concrete durability and strength [96]. While the foam may still offer benefits like improved thermal insulation, its mechanical properties might be compromised, requiring careful processing, blending with other aggregates, or surface treatments to ensure satisfactory performance in concrete applications.

The mechanism of PU foam as an aggregate in concrete involves several key aspects: it acts as a lightweight aggregate, reducing the overall density of the concrete mix and making it suitable for lightweight concrete applications. Its porous structure allows for some water absorption, influencing the water-to-cement ratio and hydration process, while also creating air voids that enhance thermal insulation [59,97]. Additionally, the smooth surface of PU foam results in a weaker bond with the cement matrix compared to traditional aggregates, though this can be improved with surface treatments. Furthermore, PU foam's low thermal conductivity significantly contributes to the insulation properties of concrete, making it ideal for energy-efficient construction [98].

### 3.1 Water absorption

The ratio of the weight of the sample increased after being immersed in water for 24 h to the weight of the dry sample stated as a percentage, known as the absorption of aggregate [99]. LWA are porous materials that absorb more water than normal aggregate [33]. The immersion method was used to determine the water absorption of PU foam aggregate. Researchers used immersion method for water absorption, the PU foam aggregate sample was submerged completely in the water-filled container, ensuring the foam was fully submerged and not floating. A wire mesh and cloth were used to hold the foam underwater, which prevented it from floating due to its compressible and porous nature [54,95]. According to Gomez-Rojo et al. [59], waste rigid PU foam has a 2–49 % water absorption range, which is 10–26 % less water absorption capacity than other LWAs such as coarse pumice, oil palm shell, and perlite waste [29]. Farhan et al. [100] reported that the water absorption of rigid waste disposal PU foam was 12.4 %. In addition, Ben Fraj et al. [54] used waste rigid PU foam from the disposal of an unshaped panel for coarse particles and showed a water absorption of 13.9 %, which is 2–78 % less water absorption capacity than other LWAs such as oil palm shell, coconut shell, ceramic waste pumice, coarse pumice, perlite waste, and waste glass [29]. The water absorption capacity of flexible and rigid waste PU foam varies depending on its structure and morphology. Rigid PU foam waste has a less porous and closed-cell structure; thus, it absorbs less water than flexible PU foam waste. However, the flexible foam showed the highest water absorption capacity due to the very porous nature of its cells, which is further accentuated by the presence of pores between the cell walls. The open-cell structure allows water to enter the foam more easily [59]. Specifying the amount of water absorption required for aggregates is essential for a concrete mix. Failure to do so can lead to issues with workability and consistency. To reduce this problem, Ben Fraj and Mounanga investigated whether PU foam waste with a water saturation condition in concrete gives better workability [54,101].

### 3.2 Sieve analysis

**Fig. 1:**
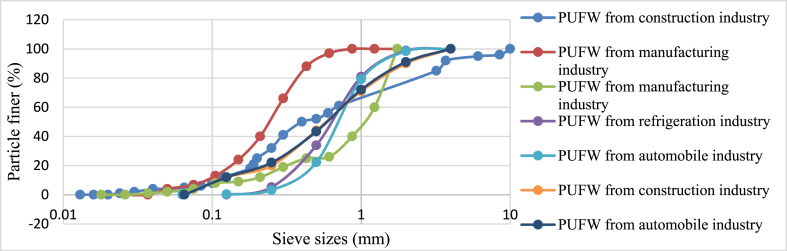
Particle size distribution of waste rigid PU foam (0–10 mm) reported in the literature [55,92,103,104].

The particle size distribution for fine and coarse aggregate is essential for lightweight concrete production. It enables quality control, informs mix design, affects workability, strength, and durability, facilitates density management, forecasts performance, and assures compliance with standards [10]. Gadea and Mounanga used rigid waste PU foam as a fine particle, a well-graded and promising material for cement concrete mixtures [55]. A well-graded aggregate mix with a balanced distribution of particle sizes makes the concrete easier to place, compact, and finish. Proper workability accelerates construction operations and reduces the requirement for excessive water content, which can develop the strength and durability of concrete [102]. Fig. 1 shows the particle size (0–10 mm) distribution of waste rigid PU foam with different industries.

### 3.3 Shape, size and texture

**Fig. 2:**
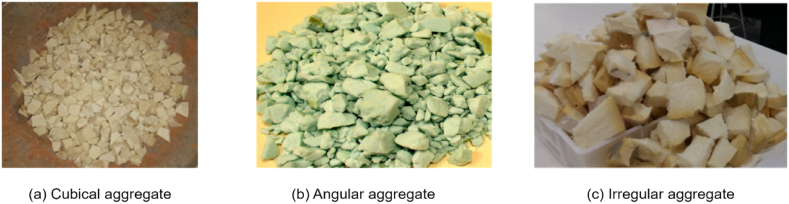
Various shapes of the PU foam waste as coarse aggregates. (a) Cubical aggregate, (b) Angular aggregate, (c) Irregular aggregate [54,59].

The shape of aggregates is an essential factor since it affects the workability of concrete [111,112]. PU foam waste typically has an irregular shape from the various industries, then is broken or crushed into regularly shaped particles with angular or rounded edges for lightweight concrete. PU foam waste is primarily yellow, grey, and white. Rigid PU foam aggregate comes from waste disposal unshaped wall panels and waste scraps [59,76]. The various shapes of PU foam aggregates, as shown in Fig. 2, include cubical (Fig. 2a), angular (Fig. 2b), and irregular (Fig. 2c) aggregates. Researchers used PU foam particle sizes ranging from 0 to 6 mm for fine aggregates and 8–20 mm for coarse aggregates in lightweight concrete [54,58]. The PU foam had a rough surface texture, which enhanced its bonding with the cement paste. Angular-shaped aggregates have sharp edges and rough surfaces, which improve the mechanical interlocking between aggregate particles and the cement paste. This leads to higher strength and better bonding in concrete [[113], [114], [115]].

Surface texture is a property that depends on how shiny, dull, smooth, or rough the surfaces of the particles are compared to each other [111,112]. Ben Fraj and Mounanga stated that PU foam waste is lightweight, naturally porous, and has a cellular structure, contributing to the LWA [54,55]. The surface texture is rough or irregular because the material is suitable for bonding with cement paste in a lightweight concrete mixture. The shape, texture, and porosity of rigid PU foam contribute to better bonding and strength in concrete than flexible PU foam. Rigid PU foam has a high closed-cell structure, resulting in higher strength, low density, and good dimensional stability. When used in concrete, the closed-cell structure of rigid PU foam allows for more excellent bonding with the concrete, resulting in increased strength and load-bearing capacity [116]. The surface texture of the rigid foam is not smooth, which improves the mechanical interlock with the concrete.

### 3.4 Bulk density

Bulk density is the mass of a given material sample divided by its total volume [117]. The bulk density of an aggregate sample depends on the shape, size distribution, and quality of the particles in the sample [[118], [119], [120]]. According to the ASTM, LWA must have a density of less than 1120 kg/m^3^ for fine aggregate and less than 880 kg/m^3^ for coarse aggregate [22]. Table 1 shows that the apparent densities or bulk densities of fine PU foam aggregate varied in the range of 26 kg/m^3^ to 430 kg/m^3^, while the real densities of fine PU foam aggregate varied in the range of 1052.7 kg/m^3^ to 2191 kg/m^3^. Rigid PU foams often have higher densities than flexible PU foams due to their less porous and closed-cell structure [59]. Higher densities improve structural integrity and load-bearing capability, making rigid PU foam suitable for applications that require strength and dimensional stability [93]. All researchers used rigid PU foam as a fine and coarse particle in concrete to develop mechanical performance. Fine rigid PU foam has a bulk density of 85 % less than traditional fine aggregate, and coarse rigid PU foam has a density of 80 % less than conventional coarse aggregate. However, compared to other LWAs made from expanded polystyrene beads, PU foam has a density that is 75 % higher [121]. Fine PU foam has a bulk density between 10 % and 80 % higher than artificially expanded perlite and exfoliated vermiculite. Bulk density is between 5 % and 35 % higher than natural rice husk ash aggregate [11,122]. Rigid PU foam has a lower density when compared to other lightweight aggregates like oil palm shells, coconut shells, and sintered fly ash [29]. However, researchers used waste rigid PU foam as an aggregate with less replacement to develop compressive strength with the required density.

**Table 1:** Physical properties of PU foam as aggregate.

Types of PU foam	Types of PU foam waste	Size of PU foam as aggregate	Apparent density (kg/m^3^)	Real density (kg/m^3^)	Water absorption 24h	References
Flexible PU foam	Waste generated from scrapped car seats.	Fine	39.8	1211.1	645.0	[59]
Semi-rigid PU foam	Waste generated from the end of its lifecycle	Fine	86.1	1378.6	333.5	[59]
Rigid PU foam	Waste comes from damage to panels used in the automotive	Fine (0–4 mm)	26 ± 2	–	–	[92]
Semi-rigid PU foam	Waste comes from the end of its life cycle	Fine (4–8 mm)	30–35	–	–	[58]
Grey colour rigid PU foam	Waste comes from the automobile components industry	Fine (0–4 mm)	59	1209	–	[105]
Rigid PU foam	Waste generated after the end of its life cycle	Fine (0–6 mm)	30	–	2–5	[57]
Rigid PU foam	Waste generated after the end of its life cycle	Fine (0–6 mm)	40	–	2–5	[57]
Rigid PU foam	Waste generated after the end of its life cycle	Fine (0–6 mm)	60	–	2–5	[57]
Rigid PU foam	Production of insulation panels from the automobile	Fine powder (0–0.5 mm)	72	1080	–	[106]
Rigid PU foam	Waste from the automotive industry	Fine (0–4 mm)	26	1210	–	[107]
Grey rigid PU foam	Waste comes from the automobile components	Fine (0–4 mm)	59	1209	–	[105]
Rigid PU foam	Waste rigid PU foam	Fine powder	62.6 ± 1.5	–	–	[108]
rigid PU foam	by-product material	Fine up to (1.75 mm)	68	–	–	[76]
Yellow rigid PU foam	Waste scrap generated when manufacturing insulation panels	Fine	141.7	1052.7	2.0	[59]
Yellow rigid PU foam	Waste material is generated from leftover or unshaped panels from factory waste.	Fine	45.5	1370.9	28.0	[59]
Yellow rigid PU foam	Waste materials are from the manufacture of insulation panels, and waste is from the factory.	Fine	56.0	1105.0	49.0	[59]
White PU foam	Waste generated from the refrigeration industry	Fine (0–1 mm)	43 ± 2	1083	–	[91]
White color rigid PU foam	Waste generated from the construction and refrigeration industries	Fine up to (4 mm)	45	1067	–	[105]
White rigid PU foam	Waste recovered from the construction and refrigeration industry	Fine (0–4 mm)	45	1067		[105]
Rigid PU foam	Waste comes from the manufacture of insulated panel	Fine Powder (0–0.5 mm)	72	1080	–	[109]
Rigid PU foam	Waste comes from damage to panels used in the building industry	Fine (0–10 mm)	45 ± 2	2191	–	[55]
Rigid PU foam	Destruction of insulation panels used in the building industry	Coarse (8–20 mm)	21	–	13.9	[54]
Rigid PU foam	Sandwich board generated waste rigid PU foam	Coarse (8–20 mm)	45 ± 2	2191	–	[56]
Rigid PU foam	PU foam insulation panel	Mixed (0.5–8 mm)	50	–	–	[110]
Rigid PU foam	PU foam insulation panel	Mixed (0.5–8 mm)	90	–	–	[110]
Rigid PU foam	PU foam insulation panel	Mixed (0.5–8 mm)	100	–	–	[110]
rigid PU foam	waste produced from insulation panel	Fine up to (1.75 mm)	72	–	–	[76]
Rigid PU foam	manufacture of insulated panel	Fine Powder (0–0.5 mm)	72	1080	–	[109]
Rigid PU foam	disposal of waste rigid PU foam	Mixed (0–15 mm)	430	–	12.4	[100]

### 3.5 SEM investigations of PU foam aggregate

**Fig. 3:**
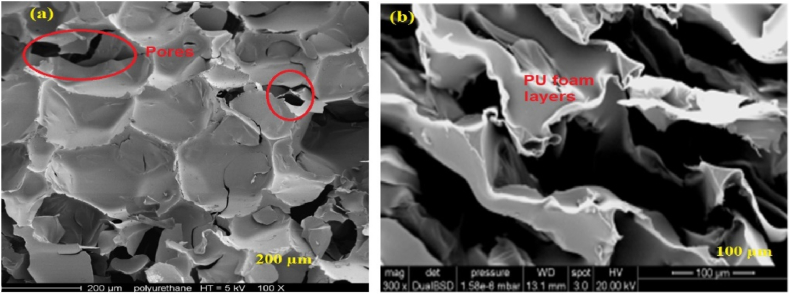
(a) & (b) Microstructure analysis of waste rigid PU foam using SEM images [55,59].

**Fig. 4:**
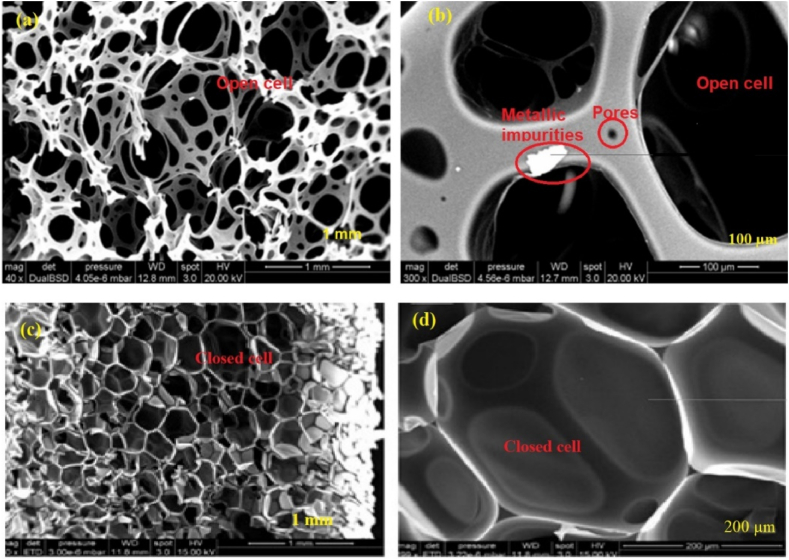
Microstructure analysis of the PU foam waste by SEM images. (a) & (b) Flexible PU foam with open cell structure. (c) & (d) Rigid PU foam with closed cell structure [59,91].

**Fig. 5:**
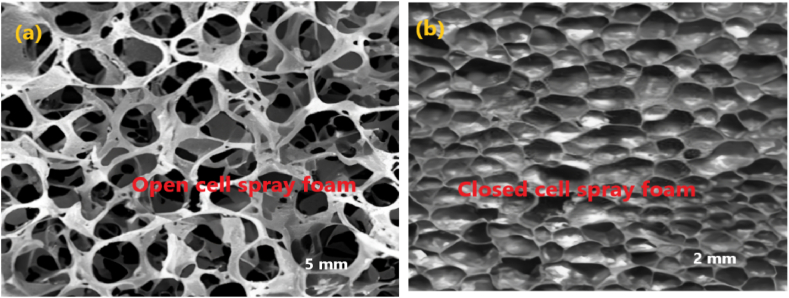
(a) & (b) Microstructure analysis of open cell and closed cell spray foam using SEM images [125].

This article also reviewed the microstructure analysis of different types of waste PU foam from different industries using scanning electron microscopy (SEM) images. SEM is a strong technology for studying the microstructure of materials under high magnification. It generates detailed photographs of the surface morphology and internal structure of PU foam particles and concrete. SEM can disclose details regarding the foam matrix pore size, shape, distribution, and connectivity [123]. The presence of pores in the cells in this particular case and PU foam layers are typical of this foam, as shown in Fig. 3 (a) & (b). SEM observation Fig. 4 (a) & (b) show that flexible PU foam has an open cell structure, and Fig. 4 (c) & (d) rigid PU foam has a closed cell structure. SEM observation shows the open porosity of foam aggregates, while the pores are cavities of about 200 μm diameter. Flexible PU foam has an open-cell structure, allowing gas to move freely between the cells because they are connected, which can improve the acoustic absorption of the material [59]. Improving the sound absorption properties by introducing textile waste into the PU foam matrix, this composite material has a cellular structure with closed pores [124]. Fig. 5 (a) and (b) show SEM images of open-cell and closed-cell spray foam, respectively.

### 3.6 Chemical elemental analysis of different PU foam waste

Table 2 shows the chemical elementary analysis of the carbon (C), nitrogen (N), hydrogen (H), oxygen (O), and calcium (Ca) components of different types of PU foam wastes that were analyzed. Carbon was the predominant component of all the types of PU foam waste [59,76]. Sulphur was not found in waste from scrapped vehicles because it is linked to elements from the actual seats, like copper or aluminium [59,103]. Smaller amounts of nitrogen and hydrogen are present in all types of PU foam waste. A significant amount of oxygen is found in PU foam waste from the automotive industry [104,107].

**Table 2:** Chemical analysis of PU foam waste.

Element	Chemical Content (%)						Reference
	C	O	N	H	Ca	Others	
Grey 1 waste PU foam	65.5	19	7.2	6.2	1	1.1	[76]
Grey 2 waste PU foam	61.4	5.5	6.8	12.4	0	13.9	[76]
Yellow 1 rigid PU foam	64.48	0	6.74	5.63	0	23.15	[59]
Yellow 2 rigid PU foam	62.06	0	6.58	5.07	0	26.29	[59]
Flexible PU foam	64.67	0	4.80	7.75	0	22.78	[59]
Semi-rigid grey PU foam	63.74	0	6.04	6.15	0	24.07	[59]
Yellow 3 rigid PU foam	63.34	0	7.28	5.58	0	23.80	[59]
Rigid PU foam	65.5	19	7.2	6.2	1	1.1	[107]
White PU foam	61.4	5.5	6.8	12.4	0	13.9	[91]
Rigid PU foam	65.5	19	7.2	6.2	1	1.1	[92]

## 4 Fresh properties of PU foam aggregate concrete

### 4.1 Workability

According to ACI 116R 2000, the slump test is the most popular method for determining the consistency of concrete, which may be done both in a lab and at the site of work [126]. It is very beneficial to find the correct mixing proportions of concrete [127,128]. Workability is an essential property of fresh concrete, which can be easily transported, stored and compacted. Generally, slump value increases with the increased water-cement ratio for LWAC.

Ben Fraj et al. [54] used waste rigid PU foam (8–20 mm) as coarse particles in LWAC and reported that the slump value ranged between 60 and 190 mm. Incorporating dry PU foam waste aggregates instead of conventional aggregates has significantly reduced slump values. The slump value of dry PU foam aggregate concrete was 60 mm, which was explained by the fact that the waste PU foam aggregate in dry conditions absorbed a high amount of water and floated during mixing because of its high porosity, which decreased the slump values and reduced workability. PU foam concrete presents several challenges in its fresh properties. Its porous structure absorbs water, leading to reduced workability and increased water demand, which can make mixing and placing more difficult [129]. To reduce this problem, pre-wetted PU foam as a coarse particle in concrete showed a higher slump value (190 mm) than dry PU foam concrete and conventional concrete. For the beneficial effect of pre-wetted PU foam, the superplasticizer was added to pre-wetted PU foam aggregate concrete to reduce water content. The slump value of pre-wetted PU foam concrete with superplasticizer ranged between 60 and 80 mm. The measured slump values for these mixes were lower than those for mixes without superplasticizer; this difference was brought about by their lower water-to-cement ratio, which was in addition to the higher cement content and higher PU-foam aggregate amount in the superplasticizer mix. The level of workability compared to without the superplasticizer mix was not significantly increased or maintained by adding the superplasticizer. The repeatability of using PU foam waste in concrete production can be challenging due to the variability in the properties of the foam, such as density, shape, size, and porosity. These inconsistencies can lead to fluctuations in the water absorption, workability, and bonding properties of the concrete mix, making it difficult to achieve uniform results. Additionally, the porous nature of PU foam increases water demand, affecting the mix consistency and strength [53,103]. To improve repeatability, stringent quality control measures are needed, including standardizing the foam waste properties, carefully controlling the water-cement ratio, and ensuring consistent mixing procedures.

In addition, Mounanga et al. [55] examined the slump value ranging between 6 and 80 mm without admixture after many trials. The size of PU foam waste is 0–10 mm, and it is used as fine aggregate, and this waste comes from destruction. It was reported that the slump value gradually decreases when waste PU foam content increases. Then, they partially replaced limestone filler with sand in PU foam concrete to give better workability because limestone filler has less water absorption and is less porous than PU foam waste. It was reported that the W/C ratio should be increased to maintain good workability because PU foam is used with fine particles and has a high porosity. Tomas et al. [58] used waste PU foam as a fine aggregate with particle sizes of 4–8 mm, without any admixture. Finely ground limestone (FGL) was added to lower the water-to-cement (W/C) ratio and reduce costs. The slump value of the concrete ranged from 70 to 210 mm, decreasing as the FGL content increased due to its higher water absorption compared to normal sand.

The inclusion of waste PU foam in concrete contributes to the workability of the concrete by improving its fluidity and flowability [130]. Waste PU foam typically has more irregular shapes and surface textures than conventional aggregates. This can result in greater particle packing and enhanced lubrication within the mix, increasing the flowability of concrete. The improved flowability makes the concrete easier to handle and place, resulting in a smoother and more uniform surface. The enhanced workability allows for better compaction, reducing the risk of voids and segregation and improving overall strength [131]. PU foam has a lower density than conventional particles used in concrete mixes; it produces lightweight concrete that is more workable due to its low mass. This lower density makes transporting, pumping, and installing easier [103].

## 5 Hardened properties of PU foam aggregate concrete

### 5.1 Density

The density of LWAC generally varies from 1400 to 2000 kg/m^3^, and for NWAC, the density is 2400 kg/m^3^. According to the ACI 213R, the density of LWAC ranged between 1350 and 1900 kg/m^3^ [39]. The density of PU foam concrete depends on various factors, such as the density of aggregate, porosity, water absorption, water content ratio, sand content, and specific gravity. Researchers studied PU foam as a complete and partial substitute for coarse and fine aggregate in concrete mixtures. Ben Fraj et al. [54] reported, using PU foam size (8–20 mm) for coarse aggregate, that the density of PU foam aggregate concrete varied between 1538 and 1699 kg/m^3^ and 27–35 % lesser than the density of NWAC and 8–64 % higher than the density of other LWAC, such as crumb rubber, expanded polystyrene beads, foundry sand, china clay sand, chromite waste, palm oil fuel ash, limestone slurry waste, and waste glass [29].

In addition, Mounanga and Gbongbon [55] used waste rigid PU foam (0–10 mm) as fine particles partially in a concrete mixture to develop compressive strength with the required density and reported that the density of PU foam concrete varies from 1261 to 1655 kg/m^3^, which is 31–47 % lower than that of NWAC. In that same study, the sand of PU foam concrete was also replaced by limestone filler. They computed the density of 1100–1679 kg/m^3^, which is nearly 30–54 % lower than the density of the NWAC. It was observed that by including limestone filler instead of sand in PU foam concrete, the density of the LWAC decreased by more than 50 % compared to the NWAC. Moreover, the density of lightweight PU concrete increased by 13 % compared to the mixes without limestone filler. Vaclavik et al. [57] investigated the waste of rigid PU foam (0–6 mm) in the concrete mixture to develop mechanical performance with the required density. They reported that the density of hardened concrete ranged between 1000 and 1200 kg/m^3^.

Tomas et al. [58] used PU foam (4–8 mm) as a replacement for fine aggregate and reported that the density of PU foam concrete ranged between 1040 and 1100 kg/m^3^, which is 55–66 % less than that of NWAC [58]. Moreover, 25 % of FGL was used in PU foam concrete instead of cement, and the reported density was 1100 kg/m^3^, which is 6 % higher than without FGL. PU foam concrete density is reduced by almost 50 % of the dead load used to construct lightweight precast elements and as a lightweight filler material. Including waste PU foam in concrete can reduce the overall density. According to the previous research report on waste PU foam in concrete, all researchers used rigid PU foam waste with a maximum density of 60 kg/m^3^, which is very low density compared to NWA and other LWAs (palm oil shell, coconut shell, sintered fly ash, ceramic waste, perlite waste and waste glass). As a result, it can easily reduce density for structural lightweight concrete as per code requirements. Polyurethane foam creates air voids in the concrete mixture. These air voids reduce the mass of the concrete while maintaining its structural integrity. This impact significantly reduces the total density of the concrete.

### 5.2 Compressive strength

Numerous investigations concentrated on the lower density of cement mixtures incorporating rigid PU foam, which impacted the static mechanical properties, particularly their strengths. As previously stated, higher PU foam contents decreased densities, leading to lower compressive strengths. Table 3 shows the mix proportions of PU foam concrete with different mix proportions, various replacement percentages, some admixture, and various types of sand. According to ACI 213R-03, the LWC has a minimum of 28 days of compressive strength of 17 MPa [39].

**Table 3:** Mix proportions of PU Foam Aggregate Concret**e**.

Size of aggregate (mm)	Mix proportions (kg/m^3^)					Volume of PUF (%)	w/c ratio	Slump (mm)	Density (kg/m^3^)	Compressive strength (MPa)	Reference
	cement	water	sand	Normal aggregate	PUF aggregate						
Sand:0–6.3 PUF: 8-20	397	220	824	–	15.1	34	0.55	190	1679	9.5	[54]
Sand:0–6.3 PUF: 8-20	415	183+ 1.4(SP)	862	–	15.8	35	0.44	80	1678	13	[54]
Sand:0–6.3 PUF: 8-20	353	156+ 1.2(SP)	734	–	20.1	45	0.44	60	1538	8	[54]
Sand:0–6.3 PUF: 8-20	397	220	824	–	15.1	34	0.55	60	1699	16	[54]
Sand:0-5 PUF: 0-10	631	441	473	–	38	17.3	0.70	80	1583	10.4	[55]
Sand:0-5 PUF: 0-10	685	411	514	–	44	17	0.60	16	1655	9.3	[55]
Sand:0-5 PUF: 0-10	761	533	0	–	55	21.8	0.70	39	1349	6.5	[55]
Sand:0-5 PUF: 0-10	699	489	0	–	73	28.2	0.70	6	1261	3.2	[55]
Sand:0-5 PUF: 0-10 LF: 0–0.3	357	422	714+ 162(LF)		23	13.1	0.81	80	1679	3.4	[55]
Sand:0-5 PUF: 0-10 LF: 0–0.3	370	437	444+ 168(LF)		47	21.2	0.81	70	1467	1.8	[55]
Sand:0-5 PUF: 0-10 LF: 0–0.3	395	467	180 (LF)		55	33.7	0.81	30	1100	1.4	[55]
PUF: 4-8	544	350	0	–	40	–	0.65	210	1050	2.79	[58]
PUF: 4-8	392	259	0	–	40	–	0.66	130	1040	2.76	[58]
PUF: 4-8	260	221	68 (FGL)	–	40		0.67	70	1070	2.60	[58]
PUF: 4-8	260	259	132 (FGL)	–	40		0.66	100	1100	2.33	[58]
PUF: 4-8	260	259	132 (FGL)	–	40		0.66	120	1080	2.69	[58]
FA: 0-5 CA: 5-25 PUF: 8-20	400	190	534	420	–	10	0.47	–	–	8.5	[56]
FA: 0-5 CA: 5-25 PUF: 8-20	400	190	534	420	–	15	0.47	–	–	7.9	[56]
FA: 0-5 CA: 5-25 PUF: 8-20	400	190	534	420	–	20	0.47	–	–	7.6	[56]
FA: 0-5 CA: 5-25 PUF: 8-20	400	190	534	420	–	25	0.47	–	–	7.4	[56]
FA: 0-5 CA: 5-25 PUF: 8-20	400	190	534	420	–	35	0.47	–	–	7.3	[56]
FA: 0-5 CA: 5-25 PUF: 8-20	400	190	534	420	–	45	0.47	–	–	6.1	[56]
FA: 0-5 CA: 5-25 PUF: 8-20	400	190	534	420	–	50	0.47	–	–	5.3	[56]

Mounanga et al. [55] first introduced waste rigid PU foam (0–10 mm) as fine particles in concrete. They investigated two mix stages: Series 1 (PU foam concrete without limestone filler) and Series 2 (PU foam concrete with limestone filler instead of sand). In series 1, they designed five various mix proportions, one for NWAC and four for PU foam LWAC. The compressive strength of NWAC was 23 MPa, whereas, in LWAC, they replaced NWA with waste PU foam (17–28 % volume fraction), and compressive strength ranged between 3.2 and 10.4 MPa. The higher volume of PU foam content in LWAC reduced compressive strength. The 17 % replacement of NWA with PU foam gave a compressive strength of 10.4 MPa. It was 54 % less than the compressive strength of NWAC. In series 2, they designed five various mix proportions: one for NWAC (reference mix), which replaced sand with limestone filler, and four for PU foam LWAC. The compressive strength of the reference mix was 24.3 MPa, whereas, in LWAC, they replaced NWA with waste PU foam (13–33 % volume fraction) and reported a compressive strength of 1.4–3.4 MPa. The 13 % replacement of NWA with PU foam and limestone filler gave a compressive strength of 3.4 MPa. It was 86 % less than the reference mix concrete. The PU foam aggregate concrete without limestone filler had better compressive strength and increased by 67 % compared to the PU foam concrete with limestone filler. Overall, the compressive strength reduced significantly as the PU foam content increased. The low compressive strength of lightweight concrete mixtures can be attributed to the PU foams large porosity, low density, and weak mechanical characteristics.

Ben Fraj et al. [54] investigated LWC by incorporating waste PU foam as coarse particles (8–20 mm) to develop the compressive strength of concrete compared to previous studies. They designed five various mix proportions, one for NWAC and four for PU foam concrete. The compressive strength of NWAC was 36 MPa, whereas LWAC replaced NWA with dry PU foam waste (35 % of the volume fraction) and reported a compressive strength of 16 MPa. In another mix proportion, pre-wetted PU foam was used in LWAC, and a compressive strength of 9.5 MPa was reported. Furthermore, the following two mixed proportions of pre-wetted PU foam concrete with superplasticizer have a compressive strength of 8 and 13 MPa. At 28 days of curing, the compressive strength of PU foam concrete decreased from 57 % to 78 % compared to the NWAC. Pre-wetting lightweight aggregates results in a 41 % decrease in compressive

strength compared to dry PU foam LWAC, which was explained by the high W/C ratio of concrete being adequate to complete cement hydration and the additional water provided by the pre-wetted PU foam particles increases porosity volume, which harms mechanical resistance. The subsequent comparison is between pre-wetted PU foam LWAC with and without superplasticizer mixes, which demonstrated the effectiveness of superplasticizer and less W/C ratio in developing the mechanical properties of PU foam concrete. In the presence of a superplasticizer, compressive strength increases by 26 %. From the all-mix proportion, the dry PU foam LWAC gave better compressive strength and increased by 35 % compared to previous studies. These compressive strengths are 32 %–87 % higher than those of other LWAC, such as 100 % replacement of bottom ash, waste glass, rice husk ash, plastic waste, foundry sand, china clay sand, palm oil fuel waste, and quarry dust [29,132].

Tomas et al. [58] used a new finely ground limestone (FGL) instead of sand with PU foam waste in the concrete matrix. The compressive strength of concrete varies between 2.33 and 2.79 MPa, which is 80 % less than conventional concrete and has low compressive strength, making it possible to use a new filler material in the LWAC segment. Vaclavik et al. [57] investigated the waste of rigid PU foam (0–6 mm) in the concrete mixture from the end of its life cycle. Different densities of PU foam in LWAC were used. They reported the compressive strength was 3.5, 5.1, and 9 MPa at 28 days after using LWAC with PU foam waste densities of 30, 40, and 60 kg/m^3^, among which the higher density PU foam gives a better compressive strength. These compressive strengths were 60 % lower than NWAC. Wang et al. [56] studied the rigid PU foam waste in concrete, which has volume fractions of 10, 15, 20, 25, 35, 45, and 50 %. After testing, Compressive strengths varied from 8.5, 7.9, 7.6, 7.4, 7.3, 6.1, and 5.3 MPa, respectively. At 28 days of curing, the compressive strength of the PU foam concrete decreased from 60 % to 79 % compared to the NWAC.

From the overall observations, the results of concrete compressive strength tests at 28 days. Incorporating PU foam waste as fine and coarse aggregate in LWAC caused a high reduction in the mechanical strength of concrete due to low mechanical characteristics and the high porosity of the PU foam. In the case of PU foam LWAC, the rupture occurred at the cementitious matrix and PU foam interfaces and in the middle of the PU foam, which creates the weak link of LWAC. On the contrary, dry PU foam LWAC nearly satisfied the structural lightweight aggregate concrete criteria defined in ACI 213 and ASTM C 330 [22,39]. This rigid PU foam lightweight concrete type is used in monolithic structures or as fill material. A possible solution for clearing these waste products is using rigid PU as a secondary raw material in the construction industry [58,59]. Rigid PU foam aggregates absorb a high amount of water due to their high porosity, increasing the drying shrinkage of concrete and thus decreasing its compressive strength. Trumble et al. [133] suggested using LWAC for floors and NWAC for columns while building a tall structure. Slabs usually represent 70 %–90 % of the total volume of concrete utilized in a structure. Furthermore, strength is not an essential issue in floor slabs, so a considerable amount of LWAC is utilized to lower the dead load of the concrete on the floors of multi-story buildings [123].

## 6 Microstructure analysis of PU foam aggregate concrete

### 6.1 SEM analysis of concrete mixture PU foam

This review article analyzed the microstructure of a cement concrete matrix with lightweight PU foam aggregate. The interfacial zone (ITZ) often significantly impactshe mechanical behaviour and transfer properties of cement-based composites [134,135]. The poor qualities of this zone are often attributed to the wall effect arising at the aggregate surface. The characteristics of ITZ vary depending on the type and porosity of the aggregates. For these reasons, ITZ in LWAC varies significantly from conventional-weight concrete [136]. Zhang et al. [137] found that using LWA on expanded clay aggregate concrete improved the ITZ between aggregate and cement paste. SEM images showed the interlocking sites given by the rough surface of LWA, which resulted in a thick and uniform interfacial zone with the cement-based mortar. Moreover, Lo and Cui [138] found that the wall effect in NWAC does not affect the ITZ of LWAC.

**Fig. 6:**
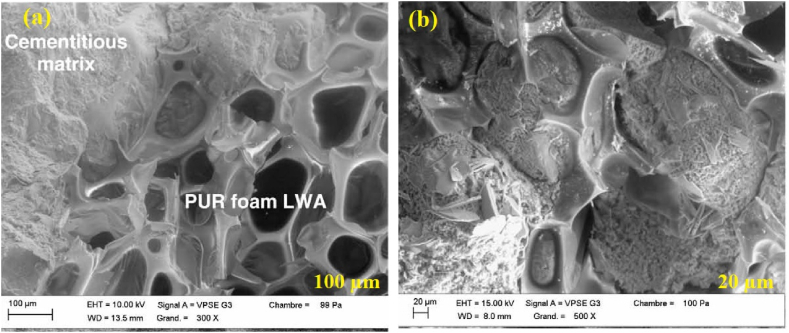
(a) & (b) SEM observation of the interface between cement paste and PU foam aggregate [54].

Kismi and Ben Fraj [54]. analyzed the microstructure of a hardened PU foam aggregate with a cementitious matrix. They showed an SEM image (Fig. 6 a & b) of LWAC incorporating waste rigid PU foam (8–20 mm) as coarse aggregate. PU foam waste comes from damaged panels used in the refrigeration and automotive industries and is then converted to coarse particles. SEM images show good adhesion between PU foam aggregate and cementitious matrix because the surface of the waste PU foam aggregate is high in porosity and not smooth. For the observation scale measured, no wall effect was observed at the interface between the PU foam aggregate and the cementitious matrix.

**Fig. 7:**
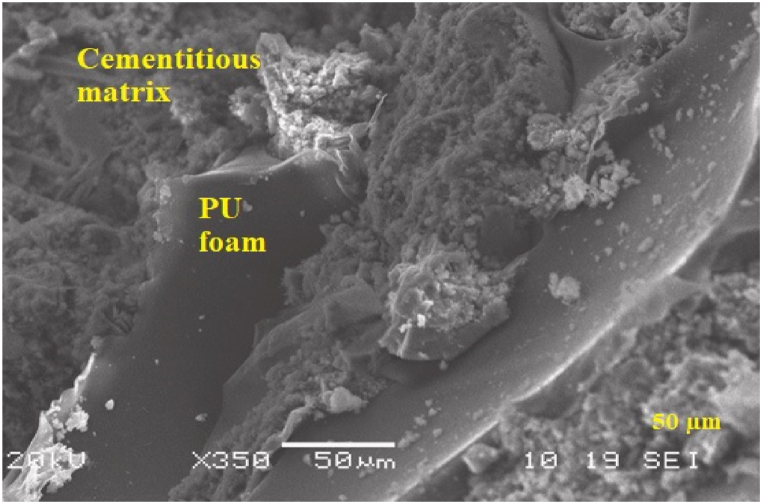
SEM observation of the interface between cementitious matrix paste and PU foam aggregate [92].

In addition, Gadea et al. [92] showed an SEM image (Fig. 7) of lightweight mortar with the incorporation of waste rigid PU foam (0–4 mm) as fine aggregate. It was reported that PU foam waste has particular flexibility that suggests the mixture has better resistance to cracks, allowing it to absorb slight structural movements without breaking up while bonding to the supporting structure. Furthermore, Alameda et al. [109] analyzed the SEM analysis of the PU foam waste reinforced with polypropylene fibre and found that the PU foam waste and gypsum matrix interlocked with the PP fibre matrix showed good adhesion between the three components. An excellent physical bond between the PU foam, gypsum matrix, and PP fibre ensured the composite stability. The bonding between PU foam and cementitious matrix can be affected by various factors, such as foam surface qualities, concrete curing conditions, and additives [139]. The compatibility of polyurethane foam with the cementitious matrix is critical for successful bonding. Polyurethane foam typically does not chemically bond to the cementitious matrix of concrete. However, polyurethane foam particles have irregular shapes or rough surfaces, allowing them to interact physically with the cement paste and aggregates [123,137]. The overall observation of the published articles is that the SEM images show good adhesion between PU foam as fine and coarse aggregate with cementitious matrix due to the waste recycled PU foam having high porous, irregular shape, surface not smooth and no wall effect was observed. SEM observations of PU foam aggregate concrete provide helpful information for understanding the composite materials microstructural characteristics and properties, which may then be used to guide its development, optimization, and application in various engineering and construction contexts.

## 7 Conclusions

The previous literature study investigated the use of waste PU foam in LWAC production. Based on the collected experimental data and previous studies, the following conclusions can be made.

• Incorporating waste PU foam into concrete helps reduce the amount of PU foam waste disposed of in landfills. Reusing the disposal of PU foam waste into concrete mixtures can reduce material costs, reduce structural load, improve workability, enhance thermal efficiency, and contribute to sustainable construction practices.
• Utilizing waste PU foam material as coarse and fine particles in concrete mixtures, thereby reducing traditional aggregate production, is an environmentally friendly solution for the traditional aggregate manufacturing industries.
• Utilizing PU foam waste with water saturation conditions in concrete develops slump value, which is suitable for developing workability in the concrete.
• Introducing waste PU foam as aggregate in concrete increases water absorption due to its highly porous structure.
• Flexible PU foam aggregate is a very low-density porous material that absorbs a higher amount of water than rigid PU foam aggregate, and rigid PU foam aggregate absorbs a higher amount of water than normal-weight aggregate. Results show that rigid PU foam has better workability than flexible PU foam.
• Using waste rigid PU foam as coarse aggregate, which is angular and cubical in shape and rough in texture, identified good bonding and better strength in all directions.
• The SEM images identified open cell structure in flexible foam, closed cell structure in rigid foam, porosity, and metallic impurities. The rigid PU foam concrete showed good adhesion between the PU foam aggregate and the cementitious matrix due to the high porous and not smooth surface.
• In all mix compositions, the dry density of the lightweight concrete with PU foam content was less than 2000 kg/m^3^ and satisfied the structural lightweight concrete criteria as per ACI 213R.
• The 28 days of compressive strengths were identified in the 5.3–16 MPa range with a density of 1538–1699 kg/m^3^ when rigid PU foam was used as coarse aggregate with conventional fine aggregate. However, compressive strength varied from 2.33 to 9 MPa with a density of 1040–1200 kg/m^3^ when rigid PU foam was used as fine aggregate and conventional coarse aggregate. When rigid PU foam is used as fine and coarse particles, it may have a compressive strength range of 1.4–10.4 MPa with a density of 1040–1679 kg/m^3^. Adding rigid PU foam aggregate to concrete decreases compressive strength due to its low density and high porosity. PU foam lightweight concrete achieved a maximum compressive strength of 16 MPa and almost satisfied the structural lightweight aggregate concrete criteria defined in ACI 213R and ASTM C 330. However, specific methods, including adding fibre and admixtures, can help enhance the mechanical qualities of concrete mixtures.

## 8 Recommendations for future research work

The following is a summary of the research gaps and some future directions for enhancing present practices.

• The results showed that adding PU foam waste as aggregate to concrete has several positive effects, such as a significant increase in workability, good bonding between the PU foam aggregate and the cement paste, and a decrease in the concrete density for lightweight structures. However, adding PU foam aggregate has specific adverse effects on concrete properties, such as decreased compressive strength and increased water absorption and porosity. Furthermore, we need to investigate further the use of PU foam waste as aggregate in concrete mixtures.
• Every year, the refrigeration, automobile, and construction industries produce several millions of tonnes of PU foam waste, resulting in fire hazards, smog, and environmental issues. PU foam waste is a good material for lightweight structures with good characteristics. So, the PU foam waste as aggregate into concrete can be studied for different types of lightweight concrete structural precast elements.
• Most researchers studied only the compressive strength and density of PU foam concrete. Further research is required for flexural strength, split tensile strength, and durability performance.
• The main drawback of PU foam aggregate concrete is its low freeze-thaw performance due to its higher water absorption capacity. However, using PU foam aggregate waste in saturation conditions and adding a water-reducing admixture before use has overcome the limitation. Further research is required to find new, effective ways to reduce the water absorption of PU foam aggregate concrete.
• The fire performance and non-destructive testing methods of PU foam aggregate concrete are not defined extensively. So, research is required to investigate the fire behaviour of PU foam lightweight concrete. However, further studies are required to investigate non-destructive testing methods.
• Future work focuses on improving the mechanical properties of the PU foam concrete mixtures by using physical and chemical admixtures.

## CRediT authorship contribution statement

**R. Roobankumar:** Writing – original draft, Methodology, Investigation, Conceptualization. **M. SenthilPandian:** Writing – review & editing, Supervision, Methodology, Investigation, Conceptualization.

## Data availability statement

The data presented in this study are available on request from the corresponding author.

## Declaration of competing interest

The authors declare that they have no known competing financial interests or personal relationships that could have appeared to influence the work reported in this paper.
